# The Aesthetics of Disillusionment: Teachers’ Narratives of “Disillusioned Transformation”

**DOI:** 10.3390/bs15070858

**Published:** 2025-06-25

**Authors:** Eunice Yin Yung Chiu, Ted Fleming

**Affiliations:** 1 Department of Educational Science, Faculty of Behavioural and Cultural Studies, Universität Heidelberg, Grabengasse 1, 69117 Heidelberg, Germany; 2 Teachers College Columbia University, 525 West 120th Street, New York, NY 10027, USA

**Keywords:** aesthetic emotions, disillusioned transformation, emotional wisdom, moral injury, systemic trauma, transformative learning

## Abstract

This article explores the emotional transformations of teachers since the pandemic, shifting from idealised passion for their profession to a more balanced, self-preserving approach to their work and mental well-being. Through four case studies of teachers from Hong Kong, Australia, and France, this paper examines how teachers navigate emotional wisdom in response to trauma and burnout and how such wisdom informs their ability to recognise when to prioritise their mental health over job prospects. The idea of disillusioned transformation will be explored: when teachers who are initially invested in a set of ideals in their roles become disillusioned and disengaged but through which find emotional balance and the resilience towards new sources of professional fulfilment. Central to transformative learning theory, this study highlights how trauma (moral injury, systemic trauma) and emotional wisdom contribute to teachers’ critical reflection and self-preservation. This article seeks to delineate the intersection between emotional wisdom, aesthetic emotions, and trauma recovery and to understand how teachers transform their professional identity in response to emotional distress, fostering a more sustainable and healthy approach to teaching.

## 1. Introduction

The COVID-19 pandemic significantly transformed the teaching profession, intensifying existing challenges and introducing new ones (Westphal et al., 2022). It created an environment in which teachers were compelled to quickly adapt to changing circumstances, such as moving between online and in-person teaching, managing different digital tools, and revising remote lesson plans (El-Soussi, 2022). Many educators reported feeling powerless in the online teaching context, as they were unable to provide the same level of personalised and emotional support that they typically offered in face-to-face classrooms (Bacher-Hicks et al., 2023).

Prolonged remote work disrupts teachers’ daily routines, offering many their first experience without the pressure of commuting to work and adhering to school schedules. Educators had to rapidly adapt their teaching methods to an unfamiliar online environment. These changes were sudden, complete, sometimes overwhelming, and all unfolding simultaneously amidst a global public health crisis. As schools transitioned between remote, hybrid, and in-person teaching, many teachers found themselves emotionally strained. Research has confirmed that issues of burnout and fatigue have long been experienced by the profession (Madigan et al., 2023). We explore how the pandemic gave teachers an unprecedented opportunity to pause, reflect, and recognise the extent of their exhaustion. Caught between institutional demands, the expectations of administrators, and the moral imperative to support their students, teachers have been under immense pressure. The physical distance imposed by the pandemic paradoxically created just enough space for them to reconsider their well-being and professional values. While much research has focused on teacher burnout and attrition, as exacerbated by the pandemic (Minihan et al., 2022; Westphal et al., 2022), this study shifts attention to the emotional transformations that teachers undergo when they reassess their professional identities and priorities.

This article introduces the concept of “disillusioned transformation” to describe this process—where teachers, once deeply committed to their profession, become emotionally detached as a form of self-protection, rather than something that is undesirable. This article interprets it as a form of “emotional wisdom” and defines it as the ability to recognise emotional limits, reassess priorities, and respond adaptively to challenging circumstances. Emotional wisdom in this sense is not only about managing emotions. We define it as understanding when the time is right for engagement and detachment and when an emotional experience has reached its aesthetic completion. We borrowed John Dewey’s concept of aesthetic emotions (Dewey, 2005). Despite the discourse on teacher well-being (Spilt et al., 2011), there remains a gap in understanding how teachers navigate “trauma” and emotional exhaustion and how that may lead to transformative changes in their professional and personal identities. This article understands that teacher burnout is a consequence of the overwhelming expectations of school leaders and of society which is experienced as a systematic trauma and an emotional labour that teachers have borne as moral injury. It seeks to understand and explore how teachers’ redefined relationships with their work are a manifestation of emotional wisdom and how this constitutes what we call “disillusioned transformation”.

The intersection of trauma, aesthetic emotions, and transformative learning will be examined in this study, hoping to contribute to ongoing discussions about the evolving nature of teaching. It offers a fresh perspective that neither romanticises nor condemns disillusionment but explores it as a complex and potentially necessary process of adaptation, especially in this post-pandemic era. Understanding this transformation is critical for policymakers, researchers, and educators in order to develop more sustainable educational environments that recognise and support the emotional realities of teachers in a volatile, uncertain, complex, ambiguous (VUCA) world (Shields, 2018).

## 2. Literature Review and Sensitizing Concepts

In this review, we introduce the main theories and concepts used in the research, occasionally illustrating them with examples from this study. These theories and concepts served as sensitizing concepts that informed the analysis of the data (Zaidi, 2022; van den Hoonaard, 1996).

### 2.1. John Dewey’s Aesthetic Experience

1. We often think of art as a collection of objects, but Dewey (Henning, 2022) argues that art is an aesthetic experience characterised by several defining qualities (Tom & Puolakka, 2021). “Phased accumulation”—an aesthetic experience builds progressively;
2. Intensity and condensation—emotions are condensed into a unified, powerful moment;
3. Resistance as growth—resistance is an essential, productive force that drives development and deepens the experience;
4. Sense of rhythm—the experiencer feels a rhythmic flow and internal momentum throughout the process;
5. Continuity and direction—seamless progressive and distinctiveness is created through a natural flow;
6. Purposeful closure—the experience concludes meaningfully by releasing built-up energy or reaching fulfilment.

Aesthetic experience is typically initiated by specific stimuli such as having a meal, glancing at a painting, etc. Aesthetic experience unfolds over time, the initial stimuli do not remain static, and they constantly evolve and blend with subsequent moments, thereby forming increasingly complex interrelations with earlier and later phases of the experience. When these evolving parts cohere in a distinct, developing unity that stands apart from the general flow of everyday experience, the resulting encounter is aesthetic. The enemies of aesthetic experiences are habitual responses or rigid abstinence and coerced submission or, in Dewey’s words, “routine, caprice and submission to convention” (Dewey, 2005, p. 40). Aesthetic experiences are not just about passive appreciation, but they involve a dynamic and emotionally charged engagement or interaction between an individual and the environment.

Emotions in aesthetic experience are not raw or chaotic; they are organised and intensified as the experience develops. Dewey argues that emotions give coherence and direction to aesthetic experiences. They are the “thread that runs through the sequence of acts and objects, knitting them together and giving them significance” (Dewey, 2005, p.44). Most importantly, they emerge when the parts of an experience flow together meaningfully. In other words, aesthetic emotion is not an addition to form but is integral to the experience’s rhythm, tension, and release; it is the emotional quality of an experience that makes it aesthetic.

### 2.2. Experience as Rupture

Disorientation is a key concept in Mezirow’s theory of transformative learning and is also central to many experiences of literature and other artistic expressions, such as paintings.

Dewey, according to Morse (2011, p. 22), has a “philosophy of rupture.” In making meaning Dewey asserted that “settled states are ‘undone,’ and this ‘undoing’ is the primary force in the meaning-making process” (Morse, p. 16). Rupture “is an important part of Dewey’s philosophy” (Morse, 2011, p. 13), and meaning in life is achieved through antagonisms and disruptions. Dewey held that discord (rupture) brings a creative force to the pursuit of meaning, even an antidote to despair (Morse, 2011). There is no such thing as a final settlement, a final definitive meaning, because every settlement introduces the conditions for a “new unsettling” (Dewey, 1938, p. 35).

Dewey is associated with the continuity of experience (Fleming, 2021, 2022b), and parallel to this continuity of experience he gives fundamental priority to the experience of rupture as part of the process of making meaning. This is a relatively unexplored concept in transformation theory, and even when it is explored (Cox & John, 2016; Scully-Russ et al., 2022; Teen et al., 2020; Watkins, 2019) there is no reference to its origins in Dewey. We learn from Mezirow (1991) that transformative learning is achieved through rupture, as in a disorienting dilemma. These unsettling experiences are akin to the puzzling experience one often finds in engagements with art. However, not all experience is a source of disequilibrium or rupture, or indeed of transformation.

Having what Alma (2020, p. 34) calls “an experience” can elicit problematic responses, and an experience may result from encountering a work of art. In the rhythm of everyday life, most of our experience is inchoate; we only become aware of what Dewey calls an experience in “problematic” situations that are beyond implicit understanding (Alma, 2020, p.35). We are no longer part of an event or situation in a taken-for-granted way. We are conscious of a rupture (Alma, 2020, p. 34). Experiences of art, according to Alma (2020), are particularly effective ways of confronting us with rupture, as frames of reference tilt out of balance by the attempt to make meaning of an aesthetic experience. It may prompt a disorienting dilemma. Alma (2020) insightfully asserts that “all art depends on friction with what we take for granted” and “breaking through our habitual ways of looking at the world” (p. 39). According to Adorno (1977), encounters with great art make “recipients lose their footing” (p. 244). The idea of being off-balance in this way may be a better way of understanding the now over-used concept of disorienting dilemma that triggers transformative learning.

### 2.3. Carol Gilligan’s Moral Injury and Systemic Trauma

Moral injury refers to the psychic harm that occurs when individuals are compelled to separate the functional aspects of their work from their emotional responses, act against their moral convictions, or remain silent in situations that demand truth-telling (Gilligan, 2014). Such injury can occur in classrooms as well, especially when teachers feel forced to act in ways that conflict with their values or find themselves unable to speak out. This invisible injury stems from suppression of their authentic voice, and the pain lies in how this suppression contradicts an individual’s moral beliefs (Gilligan, 2014). For instance, when a teacher is unable to provide individualised support to students due to large class sizes, significant learning gaps, and/or tightly packed schedules, feelings of guilt and frustration may arise. Their moral compass urges them to pause and assist those falling behind, yet institutional structures leave little space or time to act on that impulse. These moments of internal division suggest emotional rupture and conflict, compelling individuals to silence their authentic voices (Eschenbacher & Fleming, 2022). According to Gilligan (2014), this prolonged silencing amounts to trauma that becomes a recurring pattern of emotional harm embedded in institutional, cultural, and normative systems that devalue care, empathy, and emotional expression (Eschenbacher & Fleming, 2022).

The pandemic has intensified teacher burnout, yet the experience of moral injury remains underrepresented in the current literature. As emergency remote teaching progressed, many teachers witnessed the pandemic’s emotional and psychological impacts on students (Verstraeten et al., 2025). Many students seemed disengaged and isolated, and some suffered loss and trauma during that period of time, yet teachers were unable to provide the full emotional support desired by their students. Observing their students struggling, while being unable to intervene meaningfully, contributed to a deep sense of moral injury (Zembylas & Bekerman, 2018). Many teachers felt inadequate and powerless. The prolonged nature of the pandemic also added layers of constant uncertainty, contributing to significant burnout for many teachers (Westphal et al., 2022). While burnout is often associated with overwork, which has been a long-standing issue in the teaching profession (Lavery & Dahill-Brown, 2024), the pandemic added new layers of moral distress and injury. In addition, the shift to remote teaching created both geographical and emotional distance from supervisors, colleagues, and established support systems. This separation had mixed effects. On one hand, it offered teachers relief from constant evaluation and gave them greater autonomy over instructional methods, pacing, and content delivery. On the other hand, it deepened feelings of isolation and moral strain. The impact of the pandemic on teachers is therefore complex and multifaceted. While it intensified systemic trauma and moral injury, it also created space for some teachers to reflect, recalibrate, and reconnect with their passion for teaching through a deeper awareness of these systemic conditions.

### 2.4. Trauma, Disillusioned Transformation, and Emotional Wisdom

Trauma is understood here as moral injury and systemic trauma, paying particular attention to how these were intensified and made more visible during the pandemic (Khan et al., 2021). While emergency remote teaching offered teachers a degree of autonomy, it was precisely the sense of being temporarily “freed from something” that revealed the deeper constraints they had long endured. Many teachers initially welcomed the flexibility of remote teaching, only to experience persistent systemic challenges such as inequitable access to technology, rigid curricular demands, and ongoing administrative pressure, which could not be addressed through individual effort alone (Ezra et al., 2021). This marked a first layer of disillusionment: the realisation that they were embedded within a system resistant to meaningful change. It was a recognition that quickly overshadowed the early sense of freedom and autonomy.

Disillusionment can also lead to deeper reflection. Some teachers had long followed their supervisors’ directives and upheld institutional ideals, believing that their dedication would eventually be recognised through promotions, financial incentives, or opportunities for career advancement (Creagh et al., 2023). However, the pandemic disrupted this pattern of habitual compliance and prompted many to reconsider their motivations. For these teachers, the experience clarified that their true passion lay in supporting students’ academic growth and personal development. In the context of the crisis, career progression became a secondary concern. They embraced the autonomy that teaching from home provided and channelled their energy into developing innovative online teaching strategies to better engage and support their students. However, emotional wisdom can emerge from disillusionment. This article defines emotional wisdom as the capacity to recognise when and how to re-engage with emotions in order to find closure and restore relational balance. It is not simply an outcome of accumulated experience but aligns with Dewey’s concept of the unity of experience (Dewey, 2005). The flow of aesthetic emotions, including moments of epiphany and the perspective shifts that follow, are markers of the completeness of an experience (Dewey, 2005; Henning, 2022). Teachers begin to acknowledge their underlying feelings of burnout and frustration. Emotional wisdom is demonstrated by those who are able to re-engage with both their exhaustion and their passion for teaching. This reflective process leads them to reset their priorities and restore a sense of relational balance. Although the pressures from supervisors remain and institutional ideals continue to be imposed, their internal priorities change. This change involves more than courage; it provides emotional clarity, enabling them to identify what truly matters in their work (Zembylas, 2003). In the narratives shared by our informants, this process reignited their passion for teaching. A sense of relief and satisfaction followed as they recognised the need to make meaningful changes. We call this “disillusioned transformation”. It not only supports teachers’ mental health but also enhances their professional practice, allowing them to respond more effectively to students’ needs without being bound by institutional constraints. We will revisit these findings in Section 5, where they are further explored in relation to the data; they are presented here for heuristic purposes.

### 2.5. Teacher Burnout and Impacts of the Pandemic

The COVID-19 pandemic exacerbated existing pressures within schools, intensifying teacher burnout and raising global concerns about attrition and retention. While recent studies have primarily focused on emotional regulation strategies (Xiao & Tian, 2023), emotional leadership (Wang et al., 2024), and the structural factors influencing teacher burnout and well-being during the pandemic (Martínez-Líbano & Yeomans, 2023), this article shifts the analytical focus toward teachers’ inner emotional experiences and transformations involving an existential adjustment of their professional identities. Martínez-Líbano and Yeomans (2023) offer empirical insight into how the pandemic intensified emotional exhaustion among trainee teachers, and they identified psychological strain, role ambiguity, and lack of institutional support as key factors. This emotional toll has emerged as a significant predictor of burnout and potential attrition, particularly among early-career educators. Xiao and Tian (2023), in their study of Chinese EFL teachers, highlight how interpersonal emotion regulation strategies evolved during the pandemic and supported teacher well-being. Their findings underscore the importance of collective emotional coping, such as peer support and shared reflective practices, which serve as buffers against burnout and foster resilience. This aligns with broader discussions about teacher retention.

The role of emotional intelligence and leadership has also been highlighted in recent research. Xie et al. (2024) used network analysis to examine emotional intelligence in preschool teachers, revealing that self-regulation and empathy are central to emotional resilience. Complementing this, Wang et al. (2024) demonstrate that teachers’ emotional leadership positively influences student engagement, suggesting a close correlation between teacher well-being and the learning outcomes of students. This connection, in turn, contributes to professional satisfaction and retention. Unlike studies that isolate emotional traits or focus solely on external support mechanisms, this study introduces the concept of *disillusioned transformation*. This nuanced, longitudinal process describes how teachers confront moral and systemic traumas, engage in critical reflection, and ultimately achieve emotional wisdom.

Recent research has also emphasised the crucial role of technological adaptation in alleviating the challenges teachers faced during the pandemic. Chiu (2025) and Li (2024) investigate student teachers’ reflective practices in technology-enhanced environments, revealing that dialogic reflection supports both emotional processing and pedagogical growth. This suggests that such practices may enhance professional agency, reduce burnout, and should be promoted as adaptive learning habits for teachers. This aligns with our emphasis on the importance of reflecting deeply when confronted by emotionally demanding situations.

Collectively, these studies show that burnout has accelerated due to emotional demands that are intensified by the pandemic and compounded by inadequate institutional support. More importantly, emotion regulation strategies, emotional intelligence, supportive leadership, and reflective practices emerge as key protective factors that sustain teacher well-being and promote retention. The focus on cross-cultural case studies and aesthetic–emotional responses to trauma in this article offers a novel contribution to the field. Unlike existing research, this article maps how emotional distress can act as a catalyst for sustainable professional renewal and identity reconstruction.

### 2.6. Theoretical Framework: Pandemic as Aesthetic and Transformative Experience

The COVID-19 pandemic was a complex aesthetic experience marked by disruption, emotional intensity, and a reconfiguration of meaning. It offered many teachers a sudden break from the habitual life of everyday teaching. This disruption began with disturbance and triggered uncertainty and heightened emotional awareness, consistent with how Dewey contemplates the beginning of aesthetic experiences (Dewey, 2005). From a Mezirow perspective, transformative learning begins with a disorienting dilemma (Mezirow, 2000), and the pandemic played this role. As a disorienting dilemma, the pandemic disrupted educators’ assumptions about teaching, productivity, and well-being. More importantly, disruption is not an endpoint but the beginning of a transformative experience. As the pandemic unfolded, individuals moved through adaptation phases, including lockdowns, remote teaching, and anxieties about health, forcing teachers to rethink their assumptions and priorities. The connected and interrelated emotional layers shaped a continuous experiential arc; a process infused with emotions. Like Dirkx (2006, 2012) and Tisdell et al.’s (2013) transformative learning framework, emotions are essential to transformation. In this case, the emotions central to the organisation and unity of experience, such as sorrow, awe, and fatigue, are the aesthetic emotions that became meaning-making tools for these individuals, prompting reflection and value reorientation.

Echoing Dewey’s (2005) third quality of aesthetic experience, resistance is an essential and productive force that drives development, and a smooth trajectory is unlikely. When teachers faced challenging emotions such as fear, anxiety, and despair, they prompted reflection in the rhythm of pandemic life. There were waves of lockdowns and social isolation, infections and death coupled with different types of vaccines, and these internal moments of momentum, tension, and release become necessary components of the aesthetic experience. These aesthetic experiences unfold with an internal rhythm, with its own inception and resistance, bringing development and fulfilment (Dewey, 2005). Transformation theory is also a non-linear process that requires time and space to reflect. Alhadeff-Jones (2019) reminds us that time is required for learning, and often such temporal rhythms are constrained, limited, and shaped by institutionalised schedules and priorities. Teachers have managed to break through such constraints. They experienced emotional journeys through the pandemic consisting of moments of clarity, burnout, quiet joy, and realisation, which also mirrors the rhythms of aesthetic experiences and transformations.

The pandemic is not truly “over”; many are still experiencing a kind of existential culmination, and many teachers, in particular, have re-evaluated life, work, and relationships. Instead of closure, teachers have experienced a meaningful resolution, which makes the experience aesthetic. The sense of completeness or direction after reorientation corresponds to the integration of new perspectives and a redefined sense of self in the TL framework (Kreber & Cranton, 2000). For these teachers, the pandemic was a catalyst that enabled the shift that challenged their coerced submission to their supervisors and the systems. It forced them to pause their habitual and rigid flow of remaining as dutiful caretakers and instead allowed their aesthetic emotions to dye this unity of experience. In the end, it enabled them to take on the new role as emotionally wise practitioners who prioritise their own well-being. The aesthetic emotions are not just the chaotic ones; it is more than fear and grief but beauty in slowness, care, solitude, and silence (Dewey, 2005; Henning, 2022). These emotions are not just an add-on for these teachers, they are the essential substance that binds experience together in a meaningful way.

Framing the pandemic as a Deweyan aesthetic experience allows us to understand how the pandemic has the transformative potential that enables individuals to acknowledge the emotional depth amid the experiential rhythm. The pandemic is not just a public health crisis but an emergent curriculum composed of opportunities to transform, gain emotional wisdom, identity renegotiation, and professional transformation. Mezirow (2000) argues that recognising disillusionment and reorientation toward self-preservation and emotional honesty are valid forms of transformative learning and counter prevailing understandings of TL. The Deweyan framework allows us to acknowledge that the pandemic was not merely chaotic; it has its form, resistance, and rhythm that ultimately contributed to some individuals’ emotional fulfilment. Many teachers have had the space opened up by the pandemic to feel, reflect, and make peace with their limits. This realisation is central to Freire’s conscientisation, which is also crucial to the fundamentals of TL (Dirkx, 2006; Fleming, 2024; Zembylas, 2005). Instead of actions in the political sense, the conscientisation addressed here is internal. It is the awakening to the myths of professionalism and a deliberate turn toward emotional sovereignty.

## 3. Methods and Methodology

This research adopts a qualitative methodology for data collection and analysis, using five brief case studies that are supported by individual in-depth interviews, each lasting approximately one hour. This approach combines case studies and narrative inquiry within a critical narrative framework, which enables the examination of both specific lived experiences and the broader socio-political contexts that shape them (Sonday et al., 2020). This research adopts their approach because the context and the purpose align well, enabling a robust examination of teachers’ voices while acknowledging that storytelling is politically charged (Sonday et al., 2020). This approach allows us to explore both the particularities of individual lives and the broader socio-political structures that shape them. This facilitates a more meaningful discussion of trauma and transformation.

### 3.1. Case Selection

Table 1 contains the demographic details of the interviewees, outlining the similarities in their stories. The interviews were conducted via Zoom. Two individuals were contacted via a teaching network in Hong Kong, and then snowball sampling was utilized and managed to interview one teacher in Australia. The other two teachers from France were referred from another research project about European teachers’ emotions. To reduce bias, each individual was allowed to introduce only one other informant who did not work in the same school as themselves.

These five cases were selected using a homogeneous sampling strategy within purposeful sampling (Merriam & Tisdell, 2015). The selection method made isolating subtle differences easier and helps understand causal mechanisms within a controlled comparative framework (Jager et al., 2017). Contextual diversity was incorporated to reduce sampling biases. These teachers were from different countries, thus enhancing the external validity of the research, which also provided a more global or comprehensive understanding of “disillusioned transformation” (Andrade, 2018).

### 3.2. Case Study

Case study research facilitates an in-depth understanding of complex phenomena within informants’ real-life contexts (Yin, 2018). This study investigates the deeply contextual, complex, and evolving emotional transformations experienced by teachers as they navigate trauma, burnout, and identity shifts following the pandemic. Given the profound nature of these experiences, a case study approach is particularly appropriate. Yin’s (2018) methodological framework enables the exploration of “how” and “why” questions. The framework is ideal for this research, as we seek to understand how teachers develop emotional wisdom and why their professional identities transform amid systemic traumas. Merriam and Tisdell (2015) highlight the importance of purposeful sampling to support rigorous qualitative inquiry. Accordingly, this research selected cases that are information-rich, aiming for in-depth insight rather than statistical generalisability. The in-depth interviews and open sharing by informants allowed for the discovery of key themes such as disillusioned transformation and emotional trauma.

### 3.3. Narrative Inquiry

Narrative inquiry offers a powerful means to explore human experiences through the stories individuals construct and share (Bell, 2002; Ollerenshaw & Creswell, 2002). This approach reveals how individuals make meaning of their experiences over time and within social contexts, making it particularly well suited to the focus of this study. McAlpine (2016) advocates for narrative methodology due to its unique ability to capture the complexity and evolution of personal and professional identities. In this research, it enables a deep engagement with informants’ meaning-making processes while situating their stories within broader sociocultural and institutional frameworks. Kelchtermans (2009) also highlights how narrative inquiry facilitates career narratives, providing insight into teacher development and illustrating how teachers’ experiences during the pandemic have influenced their professional growth and identity construction.

### 3.4. Data Triangulation

To enhance the credibility and validity of the findings, this study employs data triangulation by drawing on multiple sources beyond interviews. In addition to in-depth one-hour interviews, field notes were taken during and after the interactions in order to capture non-verbal cues, contextual observations, and reflective impressions (Braun & Clarke, 2021). Furthermore, participants voluntarily shared personal WhatsApp messages exchanged with friends or family during the pandemic, providing candid insights into their emotional states and coping strategies. Public Instagram posts made by the informants were also included, especially those expressing strong emotions, professional struggles, and moments of resilience. Together, these diverse data sources enabled a more comprehensive and nuanced understanding of these teachers’ emotional landscapes and identity negotiations during the pandemic. Triangulation supported the development of themes while adding contextual richness and interpretive rigour to the case narratives.

### 3.5. Data Analysis

Narrative analysis focused on the stories that individuals tell about their experiences. By closely examining interviewees’ word choices, content, narrative structure, and context, this study gained an in-depth understanding of how they constructed and interpreted their realities (Creswell, 2014). Additionally, thematic analysis was employed to provide a systematic approach to managing the data, allowing us to highlight commonalities and differences across the dataset (Braun & Clarke, 2021). Table 2 outlines the initial codes, themes, and narratives derived from the interviews. Four key themes emerged, helping us understand teachers’ experiences of burnout and how these relate to the development or attainment of emotional wisdom. The integration of narrative and thematic analyses enabled a more comprehensive exploration of the data. While narrative analysis provided depth by capturing the richness of individual experiences, thematic analysis offered breadth by highlighting commonalities across cases. This combined approach facilitated a nuanced understanding of teachers’ emotional transformation.

Narrative analysis helped with understanding personal experiences and the meanings that individuals assign to them (Herman & Vervaeck, 2015). In the context of transformative learning, Cranton (2016) also pointed out that narrative inquiry and storytelling are necessary to understand individuals’ transformations. The critical narrative framework outlined by Sonday et al. (2020) combines narrative inquiry with case study methodology while emphasising power, structure, identity, and transformation. Narrative research prioritises rich, detailed accounts of lived experience over statistical generalisability, allowing for a small number of non-representative subjects to be studied in depth. This approach attends to the complexity and meaning often lost or diluted in larger samples (Riessman, 2008). It is particularly suited to researching hard-to-reach, excluded, vulnerable, or marginalized groups. Emphasising this brings individual stories to the fore and can illuminate universal themes (Riessman, 2008). Nussbaum (1992) argues that individual stories can challenge dominant norms and provoke ethical reflection, even if they are not statistically representative. When the goal is to address exclusion and injustice, representative samples become less relevant. Narrative studies of individuals can generate new insights, and through reflexive in-depth interviews and critical analysis, even a single narrative can be crucial in revealing and challenging broader frameworks (Flyvbjerg, 2006). Prioritising theoretical outcomes over statistical outcomes also supports the use of small samples in this case study and narrative methodology. Using these principles, this research implements the following steps in narrative analysis:

(1) Familiarising with the contexts of each case;
(2) Construction of narratives;
(3) Thematic coding of data;
(4) Critical coding by refining thematic coding;
(5) Conducting narrative analysis with a power and reflexivity lens.

The researchers synthesised findings on emotions, contextual factors, and theoretical insights. The first step involves understanding participants’ backgrounds, professional contexts, and broader systemic forces (such as COVID-19 disruptions and national education policies). Transcripts were read repeatedly, along with notes taken on teachers’ biographical details, institutional contexts, and socio-political influences.

The second step focused on creating coherent narratives that were both chronological and thematic. Key turning points such as moments of emotional exhaustion or burnout were identified, with relevant quotations woven into the stories.

The third step applied codes for emerging emotional themes and critical structures of power, identity, trauma, and disillusionment. Descriptive codes, such as emotional clarity and moral injury, were applied alongside critical codes like health versus student welfare and systemic pressures.

The fourth step examined how teachers’ narratives were shaped by systemic trauma, institutional expectations, and internalised ideals, focusing on the structural forces evident within the narratives.

The final step situated the findings within the transformative learning literature. From this synthesis, the concepts of disillusioned transformation and emotional wisdom emerged. This final analytic narrative integrated emotions, contextual factors, and theoretical insights.

### 3.6. Limitations and Future Research

Four of the five teachers who participated in this study were women (Theofanidis & Fountouki, 2018). While this gender distribution may reflect broader trends in the teaching profession, particularly in specific regions or educational levels, this study does not generalise findings to the global population of teachers. Instead, we aim to offer an in-depth, context-sensitive account of these individuals’ experiences. Although the sample is small, it is nonetheless valid and meaningful to represent the unique perspectives and narratives of these five participants, especially within a qualitative, interpretive framework that prioritises depth over breadth. This “gender imbalance” may be addressed in future research too.

This study focuses on the pandemic as a trigger point, but changes and developments in participants’ experiences and perspectives may continue over time. This might limit the long-term impact of the pandemic on teachers’ professional identities and well-being.

## 4. Findings: Distance Brings Clarity

The narratives shared by the five teachers reveal common threads in their emotional journeys as teachers during COVID. The most striking was the sense of relief many experienced when it seemed over. While some educators found emergency remote teaching to be a significant challenge to their careers (El-Soussi, 2022), for these teachers, it became a period of respite, allowing them to step away from the day-to-day pressures of their teaching roles. The sudden shift to remote work allowed them to distance themselves from the sources of stress at their schools, providing clarity about the underlying causes of their emotional distress.

### 4.1. The Teachers

For Beth, a secondary school English teacher in Hong Kong, the pandemic marked a pivotal moment in her career. Initially driven by her passion for teaching, she found herself burning out quickly and had been contemplating leaving her job for two years before the pandemic. Reflecting on her experience, Beth describes the intense emotional and physical toll that her job had taken on her:

*I love teaching. Or at least I thought I did. I think I do, but I would say the pandemic is the reason why I am still teaching. My school is very hierarchical; there’re lots of people politics. I am not the type to engage in all that, and so I am constantly pressured to do the chores that nobody wants. Just the semester before the pandemic hit, I was once given 200 exam papers to mark within two school days, meaning I still had to teach during that time. That same year, I was given the class teacher role because it comes with lots of administrative work and duties, and I was exhausted every day from work. I was always crying the moment I reached home, and I could not even converse with my family. I was swamped with work; it felt endless. The pandemic saved my life; it gave me a break. It freed me from classroom duties, people politics, admin work, and grading all those papers within an inhumane deadline. Before the break, I did not hate my supervisor or colleagues; I actually didn’t even realise that I was being exploited. I did whatever I was told because I could not say ‘no’ to them; that thought didn’t even cross my mind. During the pandemic, I felt like a heavy weight was lifted from me; that was when I realised I was overworked. Then I had the time and capacity to process things and realised I was very angry with the people I work with.*

The pandemic allowed Beth to step back from the daily grind, creating the space she needed to critically reflect on her situation. Her workload and the toxic work environment had affected her mental and physical well-being (Zembylas & Bekerman, 2018). The relief she felt was not just from the physical break but from the emotional distance that allowed her to process her feelings of anger and disillusionment.

Jay, a teacher in Hong Kong for seven years, also experienced a profound shift in emotional perspective during the pandemic. Like many others, he found the time away from the usual demands of teaching to be an unexpected source of relief. As the pressures of commuting, administrative tasks, and his supervising teacher role were temporarily lifted, he was able to recognise how these roles had contributed to his stress and burnout. His supervising role, which he had previously accepted as part of his teaching identity, became a point of contention once he was distanced from it. As Jay reflects:

*I was actually quite relieved, happy and relaxed during the pandemic. I was free from the pressure of waking up early or heavy traffic, I was free from admin duties, and I was free from my supervising teacher role” [What is a supervising teacher role?] “It is basically the teacher who plays the bad cop at the school. I was in charge of students’ manners and grooming, whether their hair is tied neatly, whether they wore any ear pins… that sort of things, our school cares about image and all that.*

The role of supervising teacher, which is responsible for policing students’ appearance and behaviour, was a source of discomfort and frustration that Jay had not fully recognised until it was removed. He describes how his responsibility, focused on image rather than learning or student well-being, created a sense of disconnection between himself and his students:

*I did not know that I hated these so much until I did not have to do it, it was a waste of time and irrelevant to students’ wellbeing and learning. It was the kind of thing that unnecessarily distanced teachers and students; it’s almost like the principal wanted us to look for a reason to ‘punish’ students and call that discipline. I used to think I like it because it felt powerful, but I didn’t feel worse after losing those duties, I actually felt closer to my students.*

The relief Jay experienced during the pandemic was not simply about time away from the classroom, it was also about the emotional distance it created, which allowed him to critically examine his role. He began to see how his supervisory duties as ineffective in aiding students’ learning, and it was also detrimental to his relationship with his students. His professional identity had been shaped by tasks that, in hindsight, seemed misaligned with his values of student care and educational growth. This led him to a deeper understanding of his own emotional needs as an educator, emphasising the importance of roles that prioritise meaningful connection over superficial expectations.

### 4.2. The Darkest Hour Before Dawn

Amy, a teacher in Melbourne with five years’ experience in a government-funded school, experienced profound disillusionment and frustration as the pandemic forced a rapid shift to online teaching. For Amy, the sense of disconnect between the expectations of school leadership and the realities of online teaching created a deep sense of isolation and helplessness. School leadership failed to acknowledge the depth of the issues involved in moving to online teaching:

*I wanted my students to learn well online, very soon I know it is a lie, we simply cannot put everything we do offline to a remote environment, it does not seem or sound right. I felt the problem, my colleagues did, our students did, but not my principal, not my supervisor. The ones who don’t actually have to teach can say whatever they want, they assume things kept going because it looked like it, but no, nothing was going!*

Amy’s experiences highlight an important emotional turning point, where the disconnect between the needs of her students and the decisions of the leadership became impossible to ignore. Despite recognising the detrimental effects on her students, especially those with specific learning needs like dyslexia, Amy felt powerless as school leaders remained indifferent to the challenges she and her students faced:

*My students were missing out a lot, two kids in my class are dyslexic, it is for sure most challenging for them. My supervisor did not do anything, nor did my principal, they think and I quote, ‘there is nothing to be done.*

This moment of emotional clarity was Amy’s “darkest hour” before she found a way to articulate the deeper issue: the disconnect between the priorities of leadership and the needs of teachers and students. Amy realised that the interests of school leadership and the teachers who are in direct contact with students were fundamentally misaligned:

*The problem is not online teaching, or support from teachers or the school, the problem is that the ones leading the school and the ones who are in direct contact with students have very different interests. I care about my students’ wellbeing, academic progress comes second, but the school cares about overall grades and results, they think progress is always measurable, they can’t be more wrong, it really is nothing like that in teaching.*

The pandemic created a pivotal moment in her emotional and professional growth. It was during this darkest hour that she came to realise how the values she had been striving to uphold, which include focusing on well-being and the whole-person development of students, were at odds with a school system fixated on grades and quantifiable outcomes. This deepened her frustration and also made her recognise that she could no longer rely on her supervisors for support, as she experienced the loneliness of being largely on her own to navigate the challenges of teaching.

Livia, a teacher in France, faced a deeply frustrating and emotionally taxing transition between remote and in-person teaching during the pandemic, a shift that left her feeling disconnected and unsafe. At the heart of Livia’s darkest hour was the fear and confusion that came with being forced back into in-person teaching before the pandemic was truly under control. Livia’s frustration with the lack of care from school leadership mirrored the broader issues of misalignment between teacher priorities and the decisions of authority figures:

*I told our principal that we should just stay remote until the pandemic was really over, but then she insisted. I was not worried for myself, I live with my mother-in-law, she is 90-year-old, what if I make her sick? I am sure many students face something similar too, they live with their grandparents or younger siblings. It was risky for everyone.*

While Amy was dealing with the emotional exhaustion of an ill-prepared transition to online teaching, compounded by a lack of support from school leadership, Livia was grappling with the physical risks of returning to the classroom:

*The problem was we didn’t know there was going to be a second lockdown, so I was unsure, but I did not insist further to stay at home. In retrospect, I think the government, the school, and our principal did not really care so much about teachers and students. They push for learning and resuming normal, but it was not the right time, things were not normal yet, and that was just risky for everyone. I think I see our principal differently after the whole thing, I lost respect because I think he doesn’t care about our well-being.*

Livia lost respect for her principal because of his disregard for teacher and student well-being. For both Amy and Livia, the pandemic shattered their trust in educational leadership, and they were forced to re-examine their teaching realities without the support of each other on which they once assumed they could rely. These painful realisations marked the beginning of a more self-reliant approach to teaching embedded in heightened self-awareness of their roles in the systems.

### 4.3. Time for Change

In the midst of the pandemic, these teachers’ reflections revealed a transformative shift in their professional identities and approaches to teaching. The pandemic became a catalyst for change, forcing them to reassess their roles, priorities, and relationships with students, parents, and school leadership. For each teacher, the challenging circumstances of remote learning and the upheaval of their routine prompted a fundamental redefinition of their approach to teaching, resulting in a more balanced, student-centred, and emotionally fulfilling practice. Each teacher has a different perspective shift which is unique to them in their case, and for this reason, this will be introduced separately by case.

### 4.4. Kate’s Realisation: Parent Collaboration and Personal Growth

Kate is a primary school teacher in France, and for her, the pandemic illuminated the potential for closer collaboration between the school and parents who were also working from home. She no longer viewed parents as a source of conflict or pressure but as partners in her educational efforts:

*It was easy for me during the pandemic because the parents were very helpful. My students are young, their parents had to work from home, so everyone was there. We kept everything light, but I put up progress and extra learning materials on Padlet. The parents took care of everything; the students did not suffer from learning loss I would say. Some of them even showed improvements. That made me think about the role of a teacher, perhaps it should have always been that way. Parents usually expect a lot from us, but when they know they can help as well, it seems they are willing to and are able to. I completely changed my approach with parents after the experience. I communicate openly with them, I set up goals with students, one by one based on their personalities, characters. Those goals become their goals at home too, and it was very effective, many of my students improved overall, not just their studies but their characters as well.*

Kate experienced a shift in power dynamics: isolation replaced collaborative teaching. Through empowering parents to take an active role in their children’s learning, she improved academic outcomes and strengthened the students’ personal growth, making a clear departure from traditional teacher–parent dynamics, involving the mutual benefit of working together.

### 4.5. Beth’s Empowerment: Reclaiming Autonomy and Purpose

The pandemic allowed Beth to reclaim her personal autonomy by rejecting the pressures of office politics. No longer willing to sacrifice her well-being for professional advancement, she chose to focus on building stronger relationships with her students, emphasising trust and long-term emotional investment over short-term career gains:

*I figured I should not try so hard at work, in the office, I now dare to say no. I am not sure what changed, I will still be the last person to be promoted because I don’t play their games, but at least now I don’t have to be played. I don’t have to cry from work anymore. I focus on my relationship with students, they are good kids, but they are not the very bright ones, so working on building trust and good relationship really helps them, it motivates them. It is something that takes a lot of time and efforts, but I finally have the time to do that now. That is a lot of work, but it is work that doesn’t exhaust me. This is why I said earlier, I love teaching. I am sure I do, even more now.*

Beth’s transformation from seeking approval and avoiding trouble to prioritising students’ emotional well-being illustrates a fundamental shift in her professional identity. She now sees teaching as the creation of a supportive environment where students feel safe and valued. The changing narrative involved less reliance on external validation and a greater focus on internal fulfilment.

### 4.6. Jay’s Shift in Priorities: Moving Beyond Career Advancement

Jay’s story also reflects a similar evolution, albeit through a personal loss. Prior to the pandemic, Jay was focused on career progression and building credibility. However, after losing his uncle to the virus, Jay underwent a reassessment of what truly mattered in his life. He chose to let go of the extra duties and instead concentrate on teaching and creating a positive learning environment for his students:

*I cared a lot about getting promoted, getting credibility and taking up more duties are the way to climb the ladder. It is an interesting experience for me because all along I thought that was most important to me. Well, I lost my uncle to the pandemic, I guess that made me stop and think, as well… I gave up those extra duties as soon as I could, and now I only focus on teaching, and helping my students do better. I even draw comics and personalise notes for each class to make teaching and learning more fun. When my students enjoy the lesson, they learn well and perform better in the exams. That becomes my priority now.*

During the pandemic Jay rediscovered his love for teaching and the joy of learning rather than external markers of success. His shift from careerism to student-centred learning echoes a larger theme of personal growth and clarity that came from the chaos of the pandemic.

### 4.7. Amy’s Authenticity: Teaching on Her Own Terms

Amy’s story, like Jay’s, highlights the emotional and personal growth that came from the pandemic’s challenges. She realised that the system was unlikely to change, but that she could still change herself and remain true to her values as a teacher:

*The system won’t change, the principal won’t change, but things can be changed if it starts from me. I don’t want to rush my students or pressure them; I want them to learn and blossom in their pace and based on what they are able to. I just do my own adjustments now and I don’t try to convince or negate my principal or the supervisors. I am on a permanent contract anyway, so they can’t do anything. But to myself, at least I am finally true to myself and doing something that is genuinely good for my students. I think that feeling beats anything.*

Amy’s newfound confidence and authenticity as a teacher reflect how she shifted away from systemic pressures to a deeper commitment to her students’ well-being. She chose to embrace her own teaching style and focus on individual student growth, feeling that she no longer needed to conform to the demands of the system. For Amy, this personal decision to align her teaching with her values brings a sense of fulfilment that outweighs the pressures of her institution.

### 4.8. Livia’s Reflection: Health and Well-Being Above All

Prior to COVID, health and well-being were not Livia’s primary concerns, but the pandemic forced her to reconsider her priorities, and she now places greater importance on mental and physical health, recognising that true success in teaching cannot solely be measured by academic outcomes:

*I did not care so much about health and wellbeing before. The pandemic made me think a lot about these. As a teacher, I face at least one hundred students each year, I ask myself what do I want best for them, I want them to be healthy, physically and mentally. I think that is what their families want most as well, for their children to be healthy and happy. I still try my best in class when I teach, but it does not bother me as much anymore when my students are not getting high marks and so on.*

This change in focus towards holistic care for students, prioritising their happiness and well-being over academic performance, marks a significant transformation in Livia’s teaching philosophy. She admits that she is no longer overly concerned with her students’ results:

*Maybe that does not make me a good teacher anymore, but it makes me a happy person who is able to bring happiness to my students and the school as well.*

This statement reflects the struggles that teachers feel when letting go of external expectations and instead seeking to nurture a healthy, supportive learning environment. By recognising that true success in teaching is not only defined by test scores but by the well-being and happiness of her students, Livia has embraced a more compassionate and balanced approach to education.

## 5. Discussion

### 5.1. Disillusionment: Reassessment of Moral Injuries

Critical reflection is essential for transformative learning, as it enables learners to gain new perspectives and experience a shift in their frames of reference (Cranton, 2016; Fleming, 2024; Mezirow, 2000). In this case, these teachers’ perspective shift is demonstrated by their stronger convictions in their values rather than a change in their values. Different from the other cases of transformation, this reassessment is triggered by their moral injuries, the moments where teachers’ ethical convictions about student care, equity, and meaningful learning clashed with what they were being asked to do (Zembylas & Bekerman, 2018). These moral injuries were previously tolerated in silence, but the pandemic created space and distance to gain some clarity of emotion and perspective. In this study, such injuries were not merely episodic but systemic, they are embedded in hierarchical school cultures unrealistic administrative demands, and the prioritisation of superficial metrics over genuine student development can result in what Gilligan (2014) refers to as systemic trauma. This is reflected in the five teachers’ stories.

Beth’s emotional collapse prior to the pandemic, such as crying daily after work and feeling emotionally disconnected from her family, was mislabelled as “burnout” or “stress” by herself. The interesting part is how she had been thinking about quitting for two whole years but did not take any action and still kept agreeing to the extra workload given to her. She did not have the luxury to gain clarity about these issues until the enforced pause of ERT gave her the space to reflect (Smart, 2013). What she initially interpreted as stress became exploitation, and she recognised how the school and her colleagues had disregarded her emotional labour and well-being. This process of realisation, intensified by the physical and emotional distance from her work environment, led to a re-evaluation of her commitment to institutional expectations. Her transformation began with disillusionment, an affective turning point for her.

Jay’s relief during the pandemic was not about teaching per se but about being freed from duties that served the image of the institution rather than student learning. Before the pandemic, he interpreted it as an empowerment, but when the duties were lifted, he realised the emotional and relational distance between him and his students was created by those duties. The temporary removal of this role not only brought relief but also sparked a critical reflection on the implicit values embedded in school discipline and his complicity in upholding them (Arcidiacono & Aber, 2017). His disillusionment is seen in his recognition that such practices were “a waste of time and totally irrelevant”, and he decided to forego gaining credibility for better promotion opportunities and use his time and efforts to cater for the needs of his students. This disillusioned transformation shows how moral injuries are not simply endured but become catalysts for moral clarity.

Amy’s story adds another layer to this moral dissonance; her frustration stemmed from the clearly identified disconnection between school leadership and classroom realities. Her account exposes how institutional denial (when she was told that “there is nothing to be done”) constituted a sense of abandonment for herself and especially for students with special learning needs. It was not only the workload or the crisis that was distressing but the deafness and refusal of acknowledgement from her supervisors in the face of nuanced, ethical challenges of teaching during the pandemic. Her disillusionment with leadership marked a point of rupture, a recognition that solidarity could no longer be expected from above. The moral injuries fuelled this disillusioned transformation, but this rupture sharpened her sense of autonomy and moral clarity in her teaching practice. These cases demonstrate how moral injury and disillusionment can evolve into emotionally complex but generative turning points. The distance granted by the pandemic, both emotionally and physically (via remote work), helped teachers gain emotional clarity about what they value and can no longer accept. This clarity initiated a reassessment and redefinition of their professional selves, away from institutional compliance and towards a more emotionally sustainable, student-centred ethos. It is precisely in the confrontation with betrayal and burnout that the seeds of emotional wisdom begin to emerge.

### 5.2. Emotional Wisdom: Paint in a New Light

The disillusionment experienced by these teachers implies the loss of trust in institutional systems, and the disillusioned transformation occurred not from a sense of destruction or relief but the re-entry into environments that now felt ethically compromised. Their decisions to stay as teachers came from their reignited passion for teaching, and this enthusiasm has made room for deeper emotional and moral awareness.

The accounts of Amy and Livia are especially salient here; both found themselves at odds with school leaders whose decisions reflected institutional priorities over teachers’ and students’ needs. Amy’s experience signalled a rift between educational leadership and the realities of teaching; this is more than a type of disagreement or conflict; it is an ethical rupture. Zembylas and Bekerman (2018) wrote about the “difficult knowledge” of educators and argued that institutional pressures can provoke moral disorientation and a sense of moral injury in teachers. In Livia’s story, her trust in school leadership deteriorated during the chaotic transition between online and in-person teaching, especially in her principal’s insistence on returning to the classroom. It fractured her sense of shared purpose because the school was seen as a community of care, but the decision reflected that it had other priorities over the well-being of teachers and students. The realisation marked a critical threshold in transformative learning, where the learner’s previous meaning structures collapse, urging the learner to reorient themselves morally and emotionally (Taylor, 2008).

Mezirow’s TL tends to frame critical reflection as a rational process which undermines the affective and relational dimensions of transformation (Taylor, 2017). As Zembylas et al. (2014) pointed out in their study, educators’ emotional experiences and memories are co-produced and regulated to reflect broader cultural and political discourses. This implies that teachers’ emotional experiences can catalyse both personal insight and collective consciousness. These teachers’ feelings of disillusionment and jadedness help them understand their pain not as personal failure but as systemic failure, a distinction that is critical for ethical clarity and professional growth. This sense of clarity and realisation in particular marks the emergence of emotional wisdom, a deepened understanding of one’s values in teaching, though forged through grief, betrayal, and moral disorientation, that now gains assurance within the individual. Instead of confrontations, Amy realises the importance of acting ethically on her own behalf and within her sphere of influence, and she no longer expects any systemic change from above, but she does not give up chances of making the situation better. Livia also shifts her focus from institutional loyalty to a renewed commitment to the well-being of her students. These insights are not merely outcomes of conventional professional development, they signal the transformation coming from disillusionment, which has altered these teachers’ orientation from power to care and responsibility.

The “darkest hour” was not simply about the despair these teachers felt, it was the necessary passage they had to go through as they confronted the dissonance between their values and the clash from their working conditions. This confrontation brought feelings of discomfort, anger, and frustration, but the pandemic also brought them peace because it bought them time and distance. This distance gave them the chance to orient with clarity, and the recognition of the disillusionment deepens the transformation, allowing them to move from insight to ethical stance, from critique to actions that are consistent with their own beliefs. The pain of moral injuries became the grounds for a more rooted, emotionally attuned, and conscious reflection of their identity, marking the disillusioned transformation. They became more aware and conscious about this clarity and the need to see clearly and act accordingly. This ethical commitment without drowning in the emotional baggage is where emotional wisdom is evident.

### 5.3. Disillusioned Transformation: Pathway to Resilience and Sustainability

The final stage of these teachers’ narratives marks a powerful shift, not simply a recovery from exhaustion, but a transformation shaped through emotional clarity, moral reckoning, and aesthetic re-engagement with the teaching profession (Smart, 2013; Zembylas & Bekerman, 2018). These teachers’ experiences reflect the movement from disillusionment to emotional wisdom, which is termed as “disillusioned transformation” in this article. The transition from burnout to resilience was initiated in solitude and sustained through an inward turn that allowed each teacher to reimagine what it means to teach well.

According to Mezirow (2000), TL entails a shift in one’s meaning perspectives, often prompted by a disorienting dilemma. The teachers in this study had experienced the disorienting dilemma in a complex way, through the pandemic but also the emergency remote teaching that resulted from the pandemic. The chaos imposed on the educational systems was more than a disorienting dilemma to these teachers; it was the rupture that exposed the unsustainability of their pre-pandemic professional realities. It triggered critical reflection and reassessment of long-held assumptions, initiating a process that provokes disillusionment and emotional awakening. Their previously internalised values such as performance metrics, institutional approval, and the pursuit of promotion were rendered hollow. Jay, Beth, and Amy faced the confrontation of the emotional toll of their pre-pandemic roles and experienced a disentanglement in their professional identities and the fulfilment of institutional expectations. The emergence of emotional wisdom, which prompted them to act on their ethical commitment, is an aesthetic experience itself. Dewey (2005) would single out this affective unity of doing and undergoing as key to aesthetic experience since it engages the individuals in a meaningful way.

Dewey’s concept of aesthetic experience enables a unique frame for understanding the changes and transformation that occurred in these teachers. When emergency remote teaching freed these teachers from the relentless administrative tasks and punitive structures of schooling, they began to re-engage with teaching as a creative, relational, and emotionally textured practice. Jay began drawing comics to enliven his lessons; Beth focused on trust and relationship building with her students; Kate collaborated deeply with parents. These were not utilitarian strategies but expressions of a reconfigured emotional style that is rooted in care, joy, and resonance. In Deweyan terms, it is an integration of emotion, intellect, and action, which is evidence of aesthetic emotions functioning as an organising force that rendered their teaching personally meaningful and experientially whole and complete (Dewey, 2005; Henning, 2022).

This aesthetic renewal was a result experienced by these teachers. Whether it was Amy’s frustration with inaccessible leadership, Livia’s grief over health risks, or Beth’s realisation of institutional exploitation, these teachers were at some point emotionally and ethically drained and broken. However, they did not dwell on this brokenness; the rupture helped them gain emotional and ethical clarity about their own values. It gave them courage to reject institutional complicity, and their reclamation of authority as moral agents in the classroom is also fuelled by both courage and emotional wisdom. In the TL framework, transformation is never only about content or knowledge; it is existential and deeply emotional (Cranton, 2016; Fleming, 2024). Zembylas et al. (2014) suggested that teachers’ emotional styles are pedagogical choices shaped by history, culture, and affect. Perhaps we were unable to locate how these teachers’ life experiences moulded their choices today.

These teachers are not just coping; they are reconstructing themselves through emotional wisdom. This is a powerful conscious choice not to revert to old norms, to go against the mainstream and decide to stay committed to sustainable practises that prioritise well-being, emotional authenticity, student-centred learning, and teacher–student relationships. Their aesthetic reorientation allowed them to reimagine teaching not as compliance but as an embodied, ethical, and expressive act that is in unity with who they are.

## 6. Conclusions

This study offers a timely and necessary reconsideration of how transformation unfolds in teaching, especially during and after a crisis. Drawing from the emotionally rich narratives of these five teachers across different contexts, this paper has traced a movement from emotional rupture (disillusionment) to moral clarity and, finally, to sustainable re-engagement with teaching (engagement with aesthetic experience). These trajectories do not conform neatly to the traditionally “positive” developmental shift, yet they align well with the elements of TL which specifically rely on critical theory (Fleming, 2022a). The disillusioned transformation is marked by pain, moral injury, and through reflection and transformation, a profound aesthetic reckoning with one’s professional values. These teachers gained clarity having become disillusioned, but this clarity did not lead to reconciliation with the system. Rather, it brought them away from institutional norms, but it brought them closer to their true self and toward more personally meaningful, ethically grounded, and emotionally sustainable practices.

One key implication of this study is the need to broaden the theoretical scope of TL to better understand how transformations can be rooted in disillusionment, if the person also experiences emotional clarity and acts with ethical resistance. These neglected forms of transformation are consequential and important to make sense of how teachers transform both personally and professionally after a crisis.

This study contributes to the growing discussion of moral injury in education. These teachers did not simply feel stressed or burnt out, they felt morally compromised, because they were forced to comply with institutional expectations that conflicted with their moral sense of right and wrong. Addressing such injuries, as Zembylas and Bekerman (2018) argue, requires not only personal healing but also systemic acknowledgment and the cultivation of spaces where moral repair can begin. As such, emotional and moral recovery should be seen not as private coping mechanisms but as forms of educational resistance and agency.

More importantly, the findings highlighted the value of aesthetic experience and emotional wisdom in bringing sustainable change. As Dewey (2005) reminded us, learning and living are at their most profound when they are felt, embodied, and creatively enacted. These teachers’ post-pandemic practices represent not only technical adjustments but aesthetic reorientations to the craft of teaching. These adjustments are what it means to these individuals to teach with integrity, purpose, and care in the aftermath of disruption.

This paper calls for a reimagining of transformation in TL, one that makes space for the disillusioned, the emotionally wounded, and the morally awake. This study suggests that the experience of and grappling with the possibility of burnout or emotional collapse can become fertile ground for alternative forms of transformation, which are affective, relational, and ethically charged. These transformations reflect strength and the capacity to stay in work with clarity, honesty, and humanity.

## Figures and Tables

**Table 1:** Informants’ demographic background and perspective shift.

Pseudonym	Teaching Experience	Location and School Type	Perspective Shift
Beth	5	Hong Kong; government-funded secondary school	Fulfilling supervisors’ commands → caring for students’ needs
Amy	5	Australia; government-funded high school	Prioritising students’ academic learning → socio-emotional learning
Kate	10	France; government-funded primary school	Not trusting students’ parents → reliance on parent–teacher collaboration
Livia	8	France; government-funded high school (lycée général)	Prioritising students’ academic learning → students’ well-being
Jay	7	Hong Kong; government-funded secondary school	Focus on gaining credits and getting promoted → prioritising students’ needs

**Table 2:** Mapping the thematic analysis process: from initial codes to findings.

Initial Codes	Themes	Narratives/Findings
Realisation of emotional exhaustion; reflecting on personal values	Moral injuries: concern for health vs. students’ interests; keeping schooling going vs. ensuring learning is happening	Emotional clarity, realisation of emotional labour, and processing trauma
Feeling uncertain and pointless; unsure about ERT; cannot rely on supervisors anymore	Intensified burnout and frustration: loneliness on the battlefield	Jadedness and disillusionment
Decided to make a change; students should be the primary concern; nothing else should matter more than health	Epiphanies: job prospects and promotion should not weigh more than health; educators should focus on students’ learning rather than satisfying supervisors; primary concerns between policymakers and teachers are and should be different	Emotional wisdom and disillusioned transformation

## Data Availability

The raw data supporting the conclusions of this article will be made available by the authors upon request.
